# Biochemical and clinical studies of putative allergens to assess what distinguishes them from other non-allergenic proteins in the same family

**DOI:** 10.1007/s11248-022-00316-8

**Published:** 2022-08-08

**Authors:** Kevin C. Glenn, Andre Silvanovich, Soon Goo Lee, Aron Allen, Stephanie Park, S. Eliza Dunn, Colton Kessenich, Chen Meng, John L. Vicini, Joseph M. Jez

**Affiliations:** 1 Bayer Crop Science, 700 Chesterfield Pkwy W, Chesterfield, MO 63017 USA; 2 Department of Biology, Washington University, CB 1137, One Brookings Dr., St. Louis, MO 63130 USA; 3 Allergy and Asthma Care of St. Louis, 8888 Ladue Road, Suite 105, St. Louis, MO 63124 USA; 4 Department of Chemistry and Biochemistry, University of North Carolina Wilmington, Wilmington, NC 28403 USA

**Keywords:** Protein family, Allergen, Protein structure, Skin prick test

## Abstract

Many protein families have numerous members listed in databases as allergens; however, some allergen database entries, herein called “orphan allergens”, are members of large families of which all other members are not allergens. These orphan allergens provide an opportunity to assess whether specific structural features render a protein allergenic. Three orphan allergens [*Cladosporium herbarum* aldehyde dehydrogenase (ChALDH), *Alternaria alternata* ALDH (AaALDH), and *C. herbarum* mannitol dehydrogenase (ChMDH)] were recombinantly produced and purified for structure characterization and for clinical skin prick testing (SPT) in mold allergic participants. Examination of the X-ray crystal structures of ChALDH and ChMDH and a homology structure model of AaALDH did not identify any discernable epitopes that distinguish these putative orphan allergens from their non-allergenic protein relatives. SPT results were aligned with ChMDH being an allergen, 53% of the participants were SPT (+). AaALDH did not elicit SPT reactivity above control proteins not in allergen databases (i.e., *Psedomonas syringae* indole-3-acetaldehyde dehydrogenase and *Zea mays* ALDH). Although published results showed consequential human IgE reactivity with ChALDH, no SPT reactivity was observed in this study. With only one of these three orphan allergens, ChMDH, eliciting SPT(+) reactions consistent with the protein being included in allergen databases, this underscores the complicated nature of how bioinformatics is used to assess the potential allergenicity of food proteins that could be newly added to human diets and, when needed, the subsequent clinical testing of that bioinformatic assessment.

**Trial registration number and date of registration** AAC-2017-0467, approved as WIRB protocol #20172536 on 07DEC2017 by WIRB-Copernicus (OHRP/FDA Registration #: IRB00000533, organization #: IORG0000432).

**Supplementary Information:**

The online version contains supplementary material available at 10.1007/s11248-022-00316-8.

## Introduction

The American College of Allergy, Asthma and Immunology reports that allergies affect millions of people daily around the world and are the sixth leading cause of chronic illness in the USA (ACAAI 2021). The ACAAI reported that approximately 30% of the adult population and 40% of the pediatric population in the USA have allergies, based on physician-diagnosis of clinical symptoms. Proteins that induce an allergic reaction are typically members of protein families in which a sizable proportion of the constituents are associated with allergic reactions (Ferreira et al. 2004; Hauser et al. 2010; McClain 2017). Protein allergen sequence databases, such as AllergenOnline (AllergenOnline 2021) and Comprehensive Protein Allergen Resource (COMPARE 2022), are updated annually through a peer-review process to provide a searchable list of allergens for understanding the basis of protein allergenicity. Search tools, such as FASTA or BLAST, are used to assess the relatedness of a protein of interest with sequences of proteins in an allergen database. These allergen databases provide a valuable bioinformatic resource used by developers of new sources of dietary proteins (Muraro et al. 2014; FDA 2019; De Marchi et al. 2021; Montanari et al. 2021) and genetically-modified (GM) crops (EFSA 2006, 2011; Codex 2009).

Regular updates to allergen databases often include the addition of newly identified homologs of proteins found in large protein families in which many members are known allergens (e.g., Bet v 1, tropomyosin, and profilin). Updates to allergen databases can also include the addition of proteins reported to have sufficient proof of IgE binding or biological activity (e.g., basophil activity or skin prick tests), where the protein sequences do not cluster into established allergenic/IgE-cross reactive homolog families (Kessenich and Silvanovich 2021). Proteins that do not cluster into pre-existing known allergen families may, therefore, represent new classes or families of allergens (Kessenich and Silvanovich 2021). However, some of these relatively unique allergens are themselves members of large protein families with expansive taxonomic breadth but with few, if any, other reported allergenic members. This subset of proteins listed as allergens in databases, herein called “orphan allergens”, is of interest because they might display structural feature(s) that are distinct from the vast majority of their protein relatives that are not listed in allergen databases.

In the USA, 5 to 20% of the population has an allergic (e.g., IgE-mediated) response to environmentally ubiquitous mold spores, including household and workplace dust (Simon-Nobbe et al. 2008; Twaroch et al. 2015; Williams et al. 2016). Mold sensitization is associated with the development of allergic disorders including allergic rhinitis and reactive airway diseases, such as asthma (Matsui et al. 2016). Three examples of orphan allergens in the 2020 version of the COMPARE (2022) allergen database are associated with mold allergies and come from two expansive protein families. Two examples of orphan allergen sequences are within the aldehyde dehydrogenase (ALDH) family (Achatz et al. 1995), and another three sequences (two representing isoforms of the same protein) are within the mannitol dehydrogenase (MDH) family (Schneider et al. 2006; Simon-Nobbe et al. 2006). These few examples of orphan allergens are the focus of the studies in this report because there are more than a hundred thousand ALDH homologs and thousands of MDH homologs in the universe of protein sequences (El-Gebali et al. 2019), but none of these many other homologs are reported as allergens.

The purpose of the present set of studies was to assess whether three examples of orphan allergen proteins listed in allergen databases have specific structural features distinct from the vast majority of their protein relatives that are not listed in allergen databases. Additionally, skin prick testing (SPT), a method commonly used to support a history-based clinical diagnosis of allergies (Heinzerling et al. 2013), was used to characterize the biological allergenic potential of recombinantly produced versions of these three orphan allergens to better understand the published data that was the basis for inclusion of these proteins in allergen databases. SPT reactivity of these three orphan allergens was evaluated, along with measuring SPT reactivity of commercial extracts from their source mold organisms and also relative to homologs (*Zea mays* ALDH and *Pseudomonas syringae* indole-3-acetaldehyde dehydrogenase, PsAldA) that are not listed in allergen databases.

## Methods

### Bioinformatics

Data for protein allergens were collected from the 2020 version of the Comprehensive Protein Allergen Resource database (COMPARE 2022). The database was searched against itself with BLASTP v2.11.0+ (Altschul et al. 1990) using default parameters. This output was then filtered with a high cut-off E-value threshold of 1 × 10^−1^ which was selected as it was the lowest E-value threshold that could be applied that retained all sequences with alignments from the initial BLAST search. This results in eliminating all network paths with E-score values up to the default threshold of 10, which are statistically poorly supported and are more likely to represent artifacts of the search process. The resulting BLAST networks, based on alignments between sequences, were then clustered into families using the Markov Cluster algorithm MCL-edge v14-137 (Van Dongen 2000; Enright et al. 2002; van Dongen and Abreu-Goodger 2012) following the protocol for clustering protein sequence similarity networks (van Dongen and Abreu-Goodger 2012) with an inflation value of 1.5. Networks were visualized in BioLayout v3.4 (Theocharidis et al. 2009) by importing the MCL-edge generated data and filtering 20% of the edges to allow graph separation. The final network figures were generated utilizing the Fruchterman-Reingold algorithm (Fruchterman and Reingold 1991) with a K-value modifier of 2.2, and using the built-in MCL function to auto assign cluster colors. All numbers are reported based off of the MCL-edge generated network, which may deviate slightly from the rendered figure due to the filtering of edges to allow graph separation.

### Protein expression and purification

Synthetic genes were obtained (GENEWIZ, Inc.) with codon-optimization for expression of *Cladosporium herbarum* (aka *Davidiella tassiana*) aldehyde dohydrogenase ChALDH (CAA55072.2), *Alternaria alternata* AaALDH (CAA55071.2), and *C. herbarum* mannitol dehydrogenase ChMDH (P0C0Y5.1, also known as AAO91801.1 in COMPARE (2022)) in *Escherichia coli*. Each gene was synthesized into a pET-28a construct for expression of a cleavable N-terminal His-tagged fusion protein. The resulting constructs were transformed into *E. coli* BL21 (DE3) for protein expression. Transformed *E. coli* BL21 (DE3) cells containing each construct were grown at 37 °C in Terrific Broth with 50 μg mL^−1^ kanamycin until A_600nm_ ~ 0.8. After induction with 1 mM isopropyl 1-thio-β-D-galactopyranoside (IPTG), the cells were then grown at 18 °C overnight. Following centrifugation (5000×*g* for 30 min), cell pellets were resuspended in lysis buffer (50 mM Tris, pH 8.0, 500 mM NaCl, 25 mM imidazole, 10% (v/v) glycerol, and 1% (w/v) Tween-20). Following lysis by sonication, cell debris was removed by centrifugation (12,000×*g* for 45 min), and the supernatant was loaded onto a Ni^2+^- nitriloacetic acid (NTA) column. The column was rinsed with wash buffer (50 mM Tris, pH 8.0, 500 mM NaCl, 25 mM imidazole, and 10% (v/v) glycerol) to remove unbound proteins, and the bound proteins were released using elution buffer (50 mM Tris, pH 8.0, 500 mM NaCl, 25 mM imidazole, 10% (v/v) glycerol, and 250 mM imidazole). PsAldA (*Psedomonas syringae* indole-3-acetaldehyde dehydrogenase) and ZmALDH from *Zea mays* were purified as described previously (McClerklin et al. 2018; Korasick et al. 2019). The His-tag removed proteins were further purified by size-exclusion chromatography using a Superdex-200 26/60 size-exclusion column equilibrated in phosphate buffered saline [PBS; 137 mM NaCl, 2.7 mM KCl, 10 mM Na_2_HPO_4_, 1.8 mM KH_2_PO_4_ (pH 7.4)].

For the clinical skin prick testing (SPT), fractions were pooled corresponding to the purified ChALDH, AaALDH, ChMDH, PsAldA, and ZmALDH, concentrated to 0.25—1 mg mL^−1^ and stored in PBS with 50% (v/v) glycerol. For protein crystallography, the ChALDH and ChMDH proteins were purified using a Superdex-200 26/60 size-exclusion column [equilibrated in 25 mM Hepes (pH 7.5) and 100 mM NaCl]. The purified ChALDH and ChMDH proteins were concentrated to 10 mg mL^−1^ and 7 mg mL^−1^, respectively. Protein concentrations were determined using the Bradford method, with bovine serum albumin (BSA) as a standard.

### Protein crystallography

Protein crystals of ChALDH and ChMDH were grown by the hanging drop vapor diffusion method at 4 °C. Crystals of ChALDH (10 mg mL^−1^) grew in drops of a 1:1 mixture of proteins and crystallization buffer [20% (v/v) PEG-100, 100 mM sodium/potassium phosphate, pH 6.2, 200 mM NaCl]. Crystals of ChMDH (7 mg mL^−1^) complexed with NADP^+^ formed in the crystallization condition of 25% (v/v) PEG-1500, 100 mM sodium propionate/sodium cacodylate/BIS–TRIS propane (2:1:2 molar ratio), pH 7.0, and 5 mM NADP^+^. All crystals were stabilized in cryoprotectant (mother liquor with 30% (v/v) glycerol) before flash freezing in liquid nitrogen for data collection at 100 °K. Diffraction data were collected at beamline 19ID of the Advanced Photon Source at the Argonne National Lab with HKL3000 used to index, integrate, and scale the collected data sets (Minor et al. 2006). Molecular replacement for ChALDH and ChMDH was performed using the three-dimensional structure of the human ALDH family 1 member A3 (PDB: 5FHZ) (Moretti et al. 2016) and probable NADP(H)-dependent MDH (PDB: 3GDG) (Nüss et al. 2010), respectively, in PHASER (McCoy et al. 2007). COOT (Emsley et al. 2010) and PHENIX (Adams et al. 2010) were used for iterative rounds of manual model building and refinement, respectively. Atomic coordinates and structure factors were deposited in the RCSB Protein Data Bank (PDB, www.rcsb.org) as follows: ChALDH apoenzyme (7KQV) and ChMDH·NADP^+^ (7KRG). Data collection and refinement statistics are summarized in Table 1.

**Table 1:** Summary of crystallographic statistics for ChALDH and ChMDH

Crystal	ChALDH (apoenzyme)	ChMDH·NADP^+^
Space group	I4	P2_1_
Cell dimensions	a = b = 157.2 Å, c = 164.6 Å	a = 88.87 Å, b = 119.3 Å, c = 111.2 Å; β = 94.88°
*Data collection*		
Wavelength	0.979 Å	0.979 Å
Resolution range (highest shell)	49.7–3.18 Å (3.29–3.18 Å)	31.4–2.04 Å (2.11–2.04 Å)
Reflections (total/unique)	66,054 / 33,545	268,033 /144,661
Completeness (highest shell)	99.8% (98.2%)	98.2% (85.3%)
< I/σ > (highest shell)	12.2 (2.2)	11.5 (2.6)
R_sym_^a^ (highest shell)	12.7% (75.5%)	11.2% (56.7%)
*Refinement*		
R_cryst_^b^/R_free_^c^	23.3% / 28.6%	16.0% / 17.8%
No. of protein atoms	13,484	15,923
No. of waters	–	388
No. of ligand atoms	–	1423
R.m.s. deviation, bond lengths	0.022 Å	0.007 Å
R.m.s. deviation, bond angles	1.50°	1.14°
Avg. B-factor: protein, water, ligand	86.3, -, - Å^2^	33.0, 32.9, 41.7 Å^2^
Stereochemistry: favored, allowed, outliers	96.0, 3.4, 0.6%	96.0, 3.8, 0.2%

^a^R_sym_ = Σ|I_h_−< I_h_>|/ΣI_h_, where < I_h_> is the average intensity over symmetry. ^b^R_cryst_ = Σ|F_o_−<F_c_>|/ΣF_o_, where summation is over the data used for refinement. ^c^R_free_ is defined the same as R_cryst_ but calculated using 5% of data excluded from refinement

### Clinical skin prick testing (SPT)

Individuals with clinical history of allergies (including confirmed allergy to mold) and met the inclusion and exclusion criteria (Supplemental Table 2), were eligible for enrollment as study participants. The SPT study was approved by WIRB-Copernicus (OHRP/FDA Registration #: IRB00000533, organization #: IORG0000432), and written informed consent was obtained from all study participants prior to SPT.

SPT was administered to each study participant by the Study Investigator, double-blinded to the test material in the 16 numbered vials per Test Kit (Table 2). Each test sample was applied as an epicutaneous skin prick (approximately 50 µl, 0.05 cc) using a Stallerpointe® (Trimedal, Switzerland) or comparable device to the flexor surface of each participant’s forearm. After 20 min, wheal and flare responses were measured (Supplementary Table 3). The mean wheal diameter was calculated by the addition of the maximal longitudinal (d1) and transversal (d2) diameter divided by two [(d1 + d2)/2]. SPT reactions were scored as positive when the wheal diameter was ≥ 3 mm larger than the wheal reaction to the negative control. Study data were collected on pre-defined and printed datasheets with a de-identified number that only the Study Investigator could link to all other study participant information.

**Table 2:** Summary of clinical SPT test kit samples

Test material	Test dosage
Histamine phosphate^1^	10 mg mL^−1^
Phosphate buffered saline (PBS) with 50% (v/v) glycerol	–
*Cladosporium herbarum*^1^	Commercial extract
*Cladosporium cladosorium*^2^	Commercial extract
*Alternaria alternata*^2^	Commercial extract
*Candida albicans*^2^	Commercial extract
*A. alternata* ALDH (CAA55071.2)	0.10 mg mL^−1^
	0.25 mg mL^−1^
*C. herbarum* ALDH (CAA55072.2)	0.10 mg mL^−1^
	0.25 mg mL^−1^
*C. herbarum* MDH (P0C0Y5)	0.10 mg mL^−1^
	0.25 mg mL^−1^
*Zea mays* ALDH	0.10 mg mL^−1^
	0.25 mg mL^−1^
PsAldA	0.10 mg mL^−1^
	0.25 mg mL^−1^

All samples were provided as coded and double-blinded

^1^Purchased from Stallergenes Greer, Lenoir, NC

^2^Purchased from ALK, Denmark

## Results

### Identifying orphan allergens in protein superfamilies

**Fig. 1:**
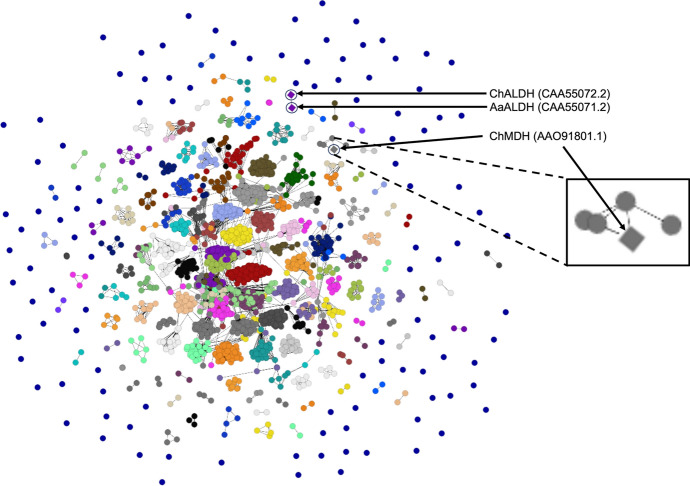
Network visualization of the 2020 COMPARE Allergen database. Visualized centrally are clusters (families) of allergens with multiple members. Around the periphery are singlet and small cluster allergens that do not share sufficient sequence similarity with large numbers of other allergens in the database. The three genes utilized in this study are circled and labeled and displayed as diamond symbols. The inset magnifies the cluster of five MDH proteins listed as allergens. From left to right they are identified in the 2020 COMPARE (2022) database as AAO91800.1, P0C0Y4.2, ACB55491.1, the utilized gene AAO91801.1, and COMPARE55. Notably the sequence for ChMDH is present in the 2020 COMPARE (2022) database under the accession AAO91801.1, and its underlying amino acid sequence is identical to that of P0C0Y5.1 which was expressed in this study

The network analysis of the 2020 version of the COMPARE (2022) allergen database (Fig. 1) reveals that the 2,248 sequences of known allergens in this database cluster into 276 protein families. Of these, the largest family, Bet v1, consists of 190 sequences (dark red colored data points located centrally in Fig. 1). Around the periphery of this network analysis of the 2020 version of the COMPARE (2022) database are the data points for singlet (105 proteins) or relatively small clusters (> 1 and ≤ 5 sequences, 94 clusters) of sequences. A significant proportion (61) of the singlet allergen clusters are “partial” sequences, and/or short peptides (< 50 amino acids in length). These peptide-length allergen sequences are less likely to generate sufficiently significant E-values for the purpose of clustering in this network analysis. Consequently, although it may be that these partial sequences and/or peptides would cluster within other families if their full-length protein sequence was available, in the absence of a high-cutoff E-value threshold they appear in this visual display of the network analysis around the periphery.

Of the remaining 44 sequences that are displayed as singlets, (and the further subset of small clusters of five proteins or less), it is noteworthy that these do not exist as evolutionary anomalies devoid of broader related proteins. Rather these sequences are often members of larger protein families, and in many cases protein superfamilies of over 100,000 representative sequences [e.g., (Pfam 2021a); ALDH].

In many instances, the sequences displayed as singlets or small clusters are a limited subset of expansive protein families in which all other members are not listed in allergen databases, i.e., orphan allergens. Two are ALDH sequences, one from *Cladosporium herbarum* (aka *Davidiella tassiana*), ChALDH (Achatz et al. 1995), and the other is from *Alternaria alternata*, AaALDH (Achatz et al. 1995). Another occurrence of orphan allergens exists within the family of MDH. One MDH orphan allergen is from *C. herbarum* (ChMDH) (Simon-Nobbe et al. 2006). Two other MDH sequences in allergen databases are from *A. alternata* (Schneider et al. 2006), although since they differ by a single amino acid, they are isoforms of the same protein. In addition to these three MDH proteins, the 2020 version of (COMPARE 2022) contains a short chain dehydrogenase (GenBank accession ACB55491.1, also known as “glucose and ribitol dehydrogenase-like protein”) and a small peptide closely related to ACB55491.1 (COMPARE database ID: COMPARE055) that shares weak identity (29%) with MDH.

### Assessing whether specific structural feature(s) of a few orphan allergens can be identified as likely allergenic epitopes

For structural comparison, the three fungal orphan allergens—ChALDH, AaALDH, and ChMDH were recombinantly produced and purified (Supplementary Figure 1). ChALDH and AaALDH are both tetrameric proteins with monomers of M_r_ ~ 54 kDa and are members of the ALDH enzyme family, which catalyze the oxidation of aldehydes to carboxylic acids and are found across multiple prokaryote and eukaryote species (Shortall et al. 2021). ChMDH is also a member of a broadly represented superfamily of multimeric enzymes (i.e., short-chain dehydrogenases/reductases (SDR) superfamily) that catalyzes the oxidation and reduction of various alcohols in multiple organisms (Kavanagh et al. 2008).

**Fig. 2:**
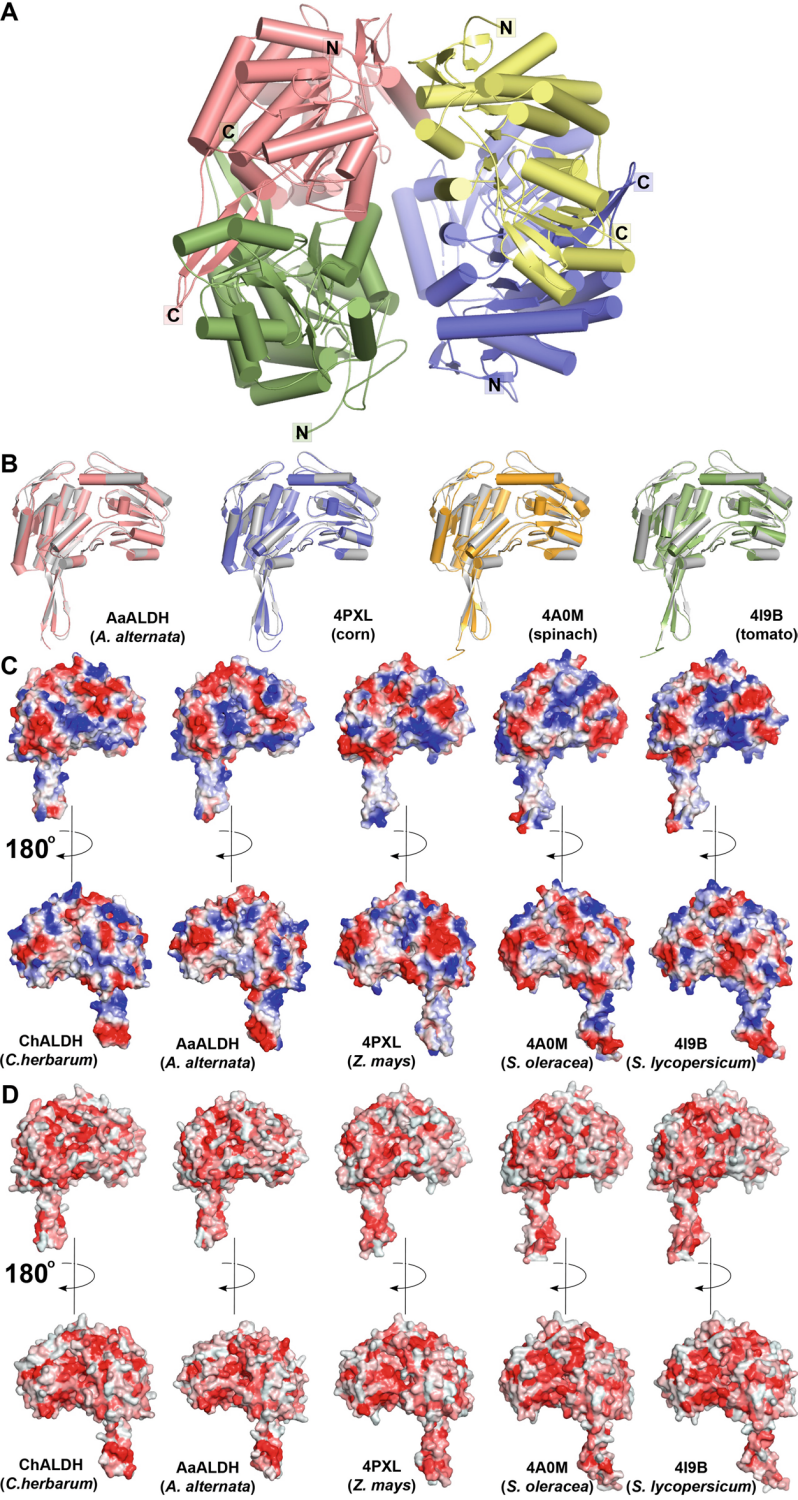
Structural analysis of ChALDH. **A** The tetrameric structure of ChALDH is shown as a ribbon diagram with each subunit differentially colored with the N- and C- termini labeled. **B** Pairwise structural comparisons of ChALDH, which is colored white in each overlay, with ALDH from *Alternaria alternata* (homology model template PDB: 5FHZ), *Zea mays* (corn/maize; PDB: 4PXL), *Spinacia oleracea* (spinach; PDB: 4A0M), and *Solanum lycopersicum* (tomato; PDB: 4I9B). Structurally related proteins were identified using the DALI server (http://ekhidna.biocenter.helsinki.fi/dali_server/). The structural alignment was performed in PyMol (Schrödinger) based on C_α_-positions. The statistics of pairwise structural comparison with ChALDH are in Supplementary Table 1A. **C** Electrostatic surface of each ALDH monomer was generated using the APBS plugin in PyMol (red = acidic; blue = basic). **D** Hydrophobicity of each ALDH monomer was calculated using the Color-h script based on the Eisenberg hydrophobicity scale in PyMol with darkest red indicating strongest hydrophobicity and white the most polar

To explore the three-dimensional structure of these orphan allergens at the molecular level, purified ChALDH and ChMDH were used for screening of protein crystallization conditions. Diffraction quality crystals of ChALDH and ChMDH were obtained, and their X-ray crystal structures were determined by molecular replacement (Table 1). The 3.18 Å resolution structure of ChALDH revealed a tetrameric structure (Fig. 2A), which corresponded with the size-exclusion chromatography analysis of the purified protein (Supplementary Figure 1). The secondary structure domains of ChALDH have high similarity to those of other ALDH, including the catalytic residues in the active site and the nucleotide cofactor binding site (Supplementary Figure 2). Each monomer unit of ChALDH retains the canonical ALDH domain organization of catalytic, NAD(P)(H) binding, and oligomerization domains (Supplementary Figure 2D). Although no ligand was bound in the ChALDH structure, amino acid residues formed interactions where a computationally docked NADP^+^ molecule is in the active site (Supplementary Figure 2F). The active site of ChALDH would form extensive van der Waals interactions with the adenine ring and nicotinamide ring of the cofactor, which is proximate to the catalytic cysteine (Cys296). Hydrogen bonds between the adenine-ribose ring and Ile161 and Lys187, as well as Trp163 and Ser241 interacting with the cofactor phosphate groups (Supplementary Figure 2F). Overall, these interactions are commonly found across the structures of the ALDH family (González-Segura et al. 2015).

The overall fold of the ChALDH monomer shares structural similarities with other ALDH family members from both prokaryotes and eukaryotes, which range in sequence identity from 32 to 57% and with root mean square deviations (r.m.s.d.) of 0.8–1.6 Å for 460–469 C_α_-atoms (Supplementary Table 1A). The human mitochondrial ALDH [PDB: 4FR8; (Lang et al. 2012)] shared the highest structural similarity with ChALDH (57% amino acid sequence identity; 0.8 Å r.m.s.d. for 469 C_α_-atoms aligned) in a DALI search of the PDB. In addition, ALDH from multiple food sources not typically associated with allergies were also identified as related to ChALDH (Supplementary Table 1A). These included the cytosolic ALDH RF2C from *Zea mays* (Korasick et al. 2019), betaine aldehyde dehydrogenase from *Spinacia oleracea* (Díaz-Sánchez et al. 2012), and ALDH from *Solanum lycopersium* (Kopečny et al. 2013).

Given that ChALDH and AaALDH are putative orphan allergens, the structural features of these enzymes were examined in comparison with other non-allergenic ALDH from common food sources. Although diffraction quality crystals of AaALDH were not obtained, a homology model was constructed for AaALDH with Swiss-Model using the three-dimensional structure of ChALDH, which shares ~ 80% amino acid sequence identity with AaALDH, as a template (Fig. 2B). The pairwise comparison between ChALDH and AaALDH indicates a conserved three-dimensional fold with similar electrostatic and hydrophobicity patterns on the surfaces of each molecule (Fig. 2C, D). Not unexpectedly, structural alignment of ChALDH with the ALDHs from maize (51% identity), spinach (43% identity), and tomato (51% identity) shows the conservation of the overall three-dimensional fold of these enzymes (Fig. 2B). In addition, there is little variation in either the surface electrostatics (Fig. 2C) or surface hydrophobicity (Fig. 2D) between ChALDH, AaALDH, and the representative ALDHs from maize, spinach, and tomato.

**Fig. 3:**
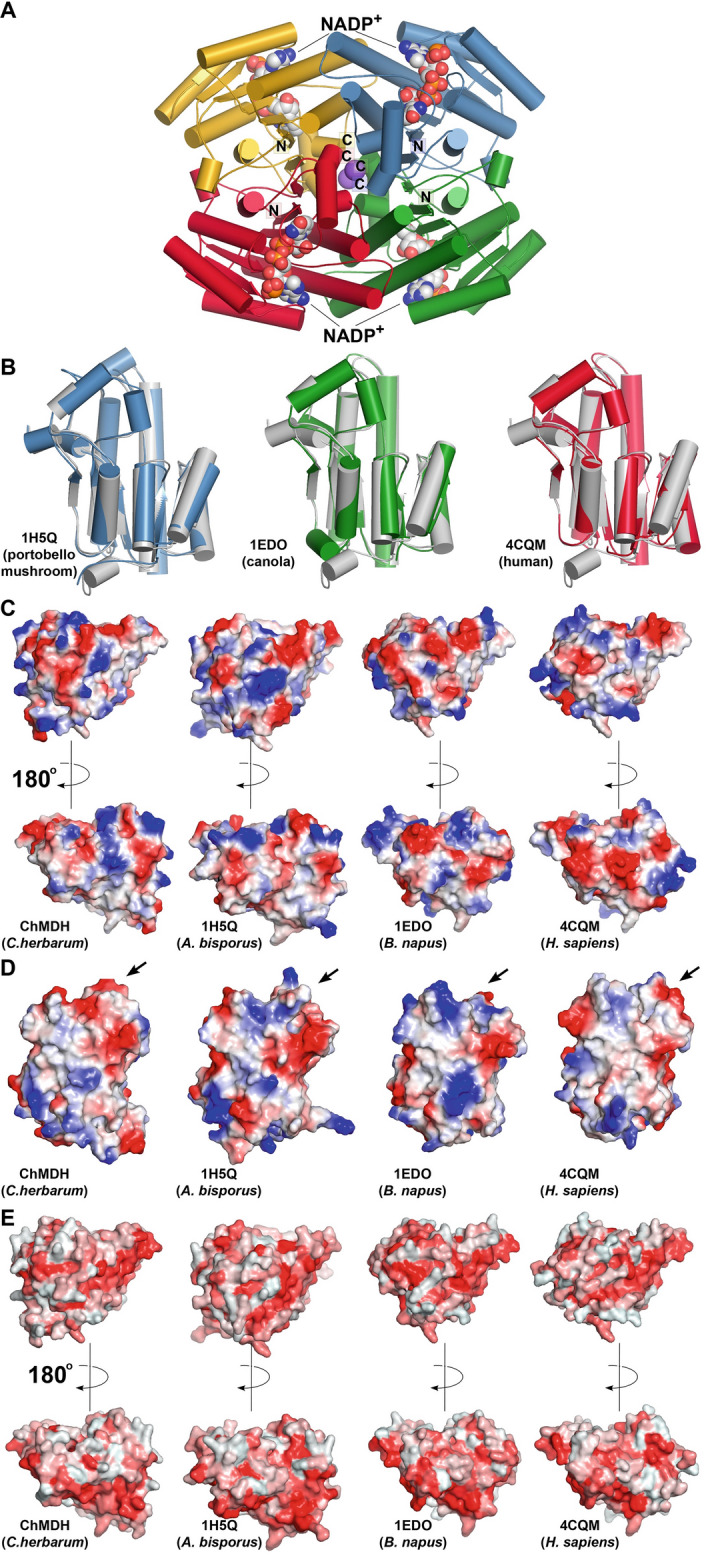
Structural analysis of ChMDH. **A** The tetrameric structure of ChMDH is shown as a ribbon diagram with each subunit differentially colored. The N- and C- termini are labeled. **B** Pairwise structural comparisons of ChMDH, which is colored white in each overlay, with structurally related SDR family members from *Agaricus bisporus* (portobello mushroom; PDB: 1H5Q), *Brassica napus* (canola; PDB: 1EDO), and *Homo sapiens* (human; PDB: 4CQM). Structurally related proteins were identified using the DALI server (http://ekhidna.biocenter.helsinki.fi/dali_server/). The structural alignment was performed in PyMol (Schrödinger) based on C_α_-positions. The statistics of pairwise structural comparison with ChMDH are in Supplementary Table 1B. **C** Electrostatic surface of each MDH monomer was generated using the APBS plugin in PyMol with red = acidic and blue = basic. **D** Hydrophobicity of each MDH monomer was calculated using the Color-h script based on the Eisenberg hydrophobicity scale in PyMol. Darkest red indicates strongest hydrophobicity to white as the most polar

The three-dimensional structure of ChMDH (the third orphan allergen selected for study) in complex with NADP^+^ was determined at 2.04 Å resolution (Fig. 3A). ChMDH is a tetrameric protein in the X-ray crystal structure (Fig. 3A) and in size-exclusion chromatographic analysis (Supplementary Figure 1). In both sequence (Supplementary Figure 3A) and three-dimensional structure (Fig. 3B; Supplementary Figure 3B-C), the monomeric unit of ChMDH is defined by the Rossmann-fold observed in multiple nicotinamide-dependent enzymes, which is conserved across members of the SDR enzyme superfamily, that includes MDH from various species (Kavanagh et al. 2008). Clear electron density for NADP^+^ in the ChMDH·NADP^+^ complex (Supplementary Figure 3C) was observed and identified the active site in the enzyme. The residues of the nicotinamide cofactor binding site in ChMDH (Supplementary Figure 3C, D) are highly conserved with other SDR family members.

The three-dimensional structure of ChMDH was used in a DALI search of the PDB to identify structurally related proteins. As expected, ChMDH showed the highest similarity with other members of the SDR enzyme family, which ranged in amino acid sequence identity from 26 to 45% with 1.1 to 1.7 Å r.m.s.d. for 237–264 C_α_-atoms (Supplementary Table 1B). Two of the structurally related SDRs are from foods common to the human diet: portobello mushroom (*Agaricus bisporus* NADP(H)-dependent MDH; PDB: 1H5Q; r.m.s.d.: 1.5 Å; 34% amino acid sequence identity; (Hörer et al. 2001) and canola (*Brassica napus* β-keto acyl carrier protein reductase; PDB: 1EDO; r.m.s.d.: 1.1 Å; 31% amino acid sequence identity; (Fisher et al. 2000)). In addition, an SDR endogenous to humans, i.e., estradiol 17β-dehydrogenase (PDB: 4CQM; r.m.s.d.: 1.5 Å; 34% amino acid sequence identity; (Venkatesan et al. 2014)) was also identified. Although the amino acid sequence of ChMDH shares low sequence identity (~ 30%) with the SDR family members from portobello mushroom, canola, and human, the pairwise structural comparisons between ChMDH and these enzymes underscores the evolutionary conservation of the protein fold in each (Fig. 3B). Comparison of the surface features of ChMDH and the representative SDRs from portobello mushroom, canola, and human reveals some variation in the electrostatic potentials of the proteins, especially in the oligomerization interfaces, (Fig. 3C) but are similar in their surface hydrophobicity (Fig. 3D).

Overall, examination of the X-ray crystal structures of ChALDH and ChMDH and a homology structure model of AaALDH did not identify any discernable putative structural epitopes that distinguish these proteins that are included in allergen databases from their protein relatives that are not listed in those databases.

### Clinical skin prick testing (SPT) of orphan allergen mold proteins ChALDH, AaALDH, and ChMDH

Although ChALDH, AaALDH, and ChMDH have been reported as allergens by different groups (Achatz et al. 1995; De Vouge et al. 1998; Schneider et al. 2006; Simon-Nobbe et al. 2006), they are unrelated to the large protein allergen families that predominate databases, such as AllergenOnline (2021) and COMPARE (2022) (Fig. 1). Instead, ChALDH, AaALDH, and ChMDH are structurally homologous to ubiquitous protein families in which their protein relatives are not listed in allergen databases (Figs. 2, 3). ChALDH, AaALDH and ChMDH were identified as putative allergens predominantly through IgE blots, with only limited, if any, additional confirmatory clinical and biological data in the published reports (Achatz et al. 1995; De Vouge et al. 1998; Schneider et al. 2006; Simon-Nobbe et al. 2006).

Typically, SPT testing is used to complement physician diagnosed allergy that is based on a clinical history of allergy-related symptoms, while serum-specific IgE has added value when assessing whole allergen extracts or particular components (Ansotegui et al. 2020). Therefore, clinical SPT studies were conducted to characterize the biological allergenic potential of recombinantly produced versions of these three orphan allergens to better understand the published allergy data that was the basis for inclusion of these proteins in allergen databases. In addition, two homologs of the three putative orphan allergen proteins that are not listed in allergen databases were included in each SPT: *Zea mays* ALDH (ZmALDH) and *Pseudomonas syringae* indole-3-acetaldehyde dehydrogenase (PsAldA). Also tested were commercial extracts from the source allergenic fungi (*C. herbarum* and *A. alternata*), a second species of *Cladosporium*, *C. cladosorium.* as well as a commercial extract of the yeast, *Candida albicans*, which is not associated with eliciting allergenic reactions [although *C. albicans* can cause inflammatory sensitization similar to SPT(+) reactions (Fukutomi and Taniguchi 2015)].

Over 18 months, 27 individuals were enrolled as study participants. Eleven of the 27 subjects elicited wheal diameters of ≥ 3 mm to the negative control [phosphate buffered saline (PBS) with 50% (v/v) glycerol] (Supplementary Table 3). This observation is consistent with clinical experience with SPT in which dermographia or other non-specific cutaneous inflammatory reactions can complicate diagnosis of clinically relevant allergic reactivity (Ansotegui et al. 2020). Therefore, for each study participant, SPT reactions to test materials were scored positive only when their wheal diameter was ≥ 3 mm larger than the wheal diameter reaction to the saline/glycerol negative control of that participant. The individual SPT results for all 27 participants to all 16 materials in the Test Kits are shown in Supplementary Table 3. Out of the 27 study participants, 19 were determined to elicit usable SPT results by showing SPT(+) reactivity to the positive control, histamine.

Of the 19 participants with interpretable SPT results, either the low and/or high dose of AaALDH and ChALDH elicited SPT(+) reactions in only one (5%) and two (11%) individuals, respectively (Table 3). This level of SPT(+) response was comparable to the level of SPT(+) responses to two related proteins, ZmALDH and PsAldA, that elicited SPT(+) reactions in one (5%) and none (0%) of the participants, respectively. However, neither ZmALDH nor PsAldA are reported in COMPARE (2022) as known allergens, unlike AaALDH and ChALDH that are both reported in this database as allergens.

**Table 3:** Summary of clinical skin prick test (SPT) results

SPT test material	Number of positive SPT reactions^1^		% Positive individuals
Histamine positive control	19		100
PBS + 50% Glycerol Negative Control	0^2^		0
*Alternaria alternata*	6		32
*Cladosporium herbarum*	6		32
*Cladosporium cladosorum*	3		16
*Candida albicans*	3		16

	Low dose (0.1 mg/mL)	High dose (0.25 mg/mL)	
AaALDH^3^	1	0	5
ChALDH^3^	1	1	11
ChMDH^3^	6	7	53
ZmALDH^4^	0	1	5
PsAldA^4^	0	0	0

SPT results for 19 participants that had validated positive responses, defined as positive to histamine. For the five purified protein tests, if a participant had a SPT(+) to either (or both) the low or high dose, they were included in the calculation of the percent of the 19 participants showing a SPT(+) response to that test material

^1^A total of 27 individuals tested, with 19 having validated SPT(+) reactions to the positive control, histamine

^2^SPT reactions were scored positive when the wheal diameter was ≥ 3 mm larger than the reaction to the negative control (phosphate buffered saline (PBS) with 50% (v/v) glycerol), therefore, by definition, all negative control tests were scored negative

^3^Listed as a putative allergen in both AllergenOnline (2021) and COMPARE (2022)

^4^Not listed as a putative allergen in both AllergenOnline (2021) and COMPARE (2022)

By comparison, 9 of the 19 participants elicited SPT(+) reactions to one or both of the commercial extracts from *A. alternata* or *C. herbarum*, which are the source organisms of AaALDH and ChALDH, respectively. Six (32%) of these study participants elicited SPT(+) reactions to both *A. alternata* and *C. herbarum*. With the exception of one subject’s SPT(+) reaction to a high dose of ChALDH, all other participants that were SPT(+) to extracts of one or both of these mold species were SPT(-) to both test doses of AaALDH and ChALDH. It is noteworthy, therefore, that the present results are not aligned with the conclusion that AaALDH and ChALDH are allergens (Achatz et al. 1995) that resulted in them being included in allergen databases.

In contrast, 10 of the 19 validated study participants (53%) showed SPT(+) reactions to ChMDH (either low and/or high doses, Table 3; Supplementary Table 3), results consistent with previous reports that ChMDH is a major allergen for *C. herbarum* allergic patients (Simon-Nobbe et al. 2006). The large number of SPT(+) responses to the two doses of ChMDH contrasted with the very limited number of SPT(+) reactions to the two ALDH proteins, that are listed in allergen databases. Interestingly, the number of study participants eliciting SPT(+) reactivity to ChMDH was the largest number of SPT(+) reactions of all test materials, including the extract from its source fungal species, *C. herbarum*.

Commercial extracts from *C. cladosorum* and *C. albicans* each produced SPT(+) reactions in three (16%) of the 19 participants. *C. cladosorum* is associated with clinical allergies (Simon-Nobbe et al. 2008), while the yeast, *C. albicans*, although not associated with elicitation of clinical allergies, can cause inflammatory sensitization consistent with SPT(+) reactions (Fukutomi and Taniguchi 2015).

## Discussion

### How are protein allergens classified into families?

Many of the proteins that induce allergic reactions can be classified into families according to structural similarities that explain cross-reactivity. For instance, in oral allergy syndrome individuals sensitized by respiratory exposure to the Bet v 1 protein found in birch pollen, allergic symptoms can be elicited upon exposure (either respiratory or digestive) to cross-reacting proteins from other sources (Biedermann et al. 2019). Nearly 30 major groups of cross-reactive proteins have been identified; pathogenesis-related proteins such as Bet v 1, enzymes (e.g., proteases, glycolytic enzymes), and others (e.g., transport proteins, protease inhibitors, regulatory proteins, structural and storage proteins) (Ferreira et al. 2004; Hauser et al. 2010; McClain 2017).

Unlike the cross-reactive allergens where many protein family members are allergenic, the present study defines orphan allergens as unique members of large protein families in which all other members are not included in allergen databases. The aldehyde dehydrogenase (ALDH) family of proteins (PF00171) in PFAM v34.0 (Pfam 2021b) was shown in 2019 to encompass 117,129 sequences stemming from 8467 species (El-Gebali et al. 2019). Similarly, the mannitol dehydrogenases (MDH) family (PF01232) in PFAM v34.0 (http://pfam.xfam.org/) is also large, encompassing 6035 sequences stemming from 3467 species (El-Gebali et al. 2019). However, only a small number of ALDH and MDH family members (Achatz et al. 1995; Schneider et al. 2006; Simon-Nobbe et al. 2006; Nakazawa et al. 2007; Cui et al. 2016; Huerta-Ocampo et al. 2020) are listed in allergen databases. One example of an ALDH listed as an allergen is from *C. herbarum* (aka *D. tassiana*), ChALDH (Achatz et al. 1995), and the other is from *A. alternata*, AaALDH (Achatz et al. 1995). ChALDH and AaALDH share 80% identity and 94% similarity spanning their complete lengths. In addition to ChALDH and AaALDH, three other members of the ALDH family are described as allergens in publications (Nakazawa et al. 2007; Cui et al. 2016; Huerta-Ocampo et al. 2020), however these three are not currently included in allergen databases, such as COMPARE (2022).

In the large family of MDH proteins, one example of an orphan allergen is sourced from *C. herbarum* (ChMDH) (Simon-Nobbe et al. 2006). Two other MDH orphan allergen examples are from *A. alternata* (Schneider et al. 2006), although since these two sequences differ by a single amino acid, they are isoforms of the same protein. In addition to these three MDH proteins, the 2020 COMPARE (2022) database contains a short chain dehydrogenase (GenBank accession ACB55491.1, also known as “glucose and ribitol dehydrogenase-like protein”) and a small peptide closely related to ACB55491.1 (COMPARE database ID: COMPARE055) that share weak identity (29%) with MDH.

The working hypothesis for this study was that the three orphan allergens, ChALDH, AaALDH and ChMDH, possess unique structural feature(s) that serve as IgE epitopes that are absent in their more prevailing protein relatives that are not listed in allergen databases. The IgE binding structures could be associated with either a sequential uninterrupted amino acid string that is elusive to identify using primary sequence-based bioinformatic methods, or as a discontinuous distribution throughout the larger protein sequence (McClain 2017).

### What structure characteristics are needed for a protein to be an allergen?

The inclusion of ChALDH, AaALDH and ChMDH in allergen databases is fitting for further assessment, as in the present studies, because closely related proteins that are not included in allergen databases are present in molds, such as *Penicillium camemberti*, *Botrytis cinerea*, and *Baudoinia panamerican*, that have a long history of consumption of foodstuffs and/or from the environment (Simon-Nobbe et al. 2008; Twaroch et al. 2015; Williams et al. 2016). The aforementioned three organisms contain genes that encode ALDH and MDH proteins that have 72–90% identity with ChALDH, AaALDH and ChMDH but have not been identified as allergens themselves. The high levels of identity observed in apparently non-allergenic homologs of ALDH and MDH found *B. cinerea, P. camemberti*, and *B. panamericana* portend not only retained structure in the protein core but also on the protein surface, the region of allergenic proteins that most typically display IgE-binding domains.

By comparison, surface similarity accounts for the cross reactivity among Bet v 1 group allergens, for instance soybean Gly m 4 displays 47% identity and 60% surface similarity with Bet v 1 (Jenkins et al. 2005). Given the high level of identity of *B. panamericana* ALDH and MDH with ChALDH and ChMDH, 90% and 87% respectively, one could reasonably expect sufficient levels of surface similarity to support cross-reactive IgE-binding. In such a scenario, ALDH and MDH should, presumably, not be orphan allergens but be members of a protein family with additional, if not plentiful, cross-reacting allergens.

Diffraction quality crystals of ChALDH and ChMDH were obtained, and their X-ray crystal structures were determined by molecular replacement. At the 2.04 to 3.18 Å resolution structure of ChALDH and ChMDH, respectively, no discernable putative structural epitopes are evident that distinguish these orphan allergens from their more prevailing protein relatives that are not listed in allergen databases. Similarly, although diffraction quality crystals of AaALDH were not obtained, a homology model constructed for AaALDH with Swiss-Model using the three-dimensional structure of ChALDH, also did not identify any discernable putative structural epitopes that are distinct from protein relatives that are not listed in allergen databases.

### How are allergens and allergies assessed clinically?

Clinical diagnosis of allergy requires multiple lines of evidence, with the most important coming from physician-diagnosed allergy based on a clinical history of allergy-related symptoms. Skin prick testing (SPT) and serum IgE testing are the most frequently used clinical laboratory tools (Muraro et al. 2014). SPT testing is typically used to complement physician diagnosis of a history of allergy-related symptoms, while serum-specific IgE helps to assess whole allergen extracts or specific components (Ansotegui et al. 2020). Although these types of lab tests can identify “sensitization”, a positive result is insufficient, by itself, to diagnose clinical allergy (Sicherer and Sampson 2018), corroborating the importance of adequate clinical history.

SPT is known to be highly variable, even under the best controlled conditions (Hamilton and Adkinson 2003; Carr et al. 2005; Simon-Nobbe et al. 2008; Ansotegui et al. 2020). One source of SPT variability is that several types of SPT devices and reagents provide different degrees of sensitivity and specificity (Carr et al. 2005). A second source of SPT variability is associated with differences in cutaneous reactivity across study participants, including dermographia and non-specific local inflammatory reactions (Ansotegui et al. 2020). A third challenge with SPT studies is that many medications can cause false negative SPT reactions including: H-2 antagonists, such as famotidine used for dyspepsia; tricyclic anti-depressants, such as amitriptyline; topical corticosteroids, such as hydrocortisone; and local anesthetics, such as lidocaine (Ansotegui et al. 2020). Although the candidates in the present study were asked to discontinue use of antihistamines, such as diphenhydramine and loratadine, they were not asked to discontinue these other widely used medications. Furthermore, it is understandable that participants would not be aware that some of their other medications, such as over-the-counter sleep-aids, contain antihistamines. A fourth source of variability in SPT reactivity, especially to some of the positive controls used in this study, is that significant variability exists between commercial suppliers of mold extracts in Europe and the USA, and no standardized extracts are available (Simon-Nobbe et al. 2008). A number of these documented sources of SPT variability most likely contributed to the variability in responses to the positive histamine control. This variability is also likely the reason that, even though all participants in the present study had confirmed clinical mold allergy (including prior positive SPT reactivity to commercial extracts of *C. herbarum* and *A. alternata*), only 10 of the 19 participants with usable reactivity to the positive control, histamine, elicited a positive SPT reaction to the batches of commercial mold extracts used during the conduct of this study.

In the present study, to facilitate interpretation of SPT responses, negative controls were used to guide clinical reading of reactivity to the test materials. It was observed that 11 of the 27 subjects elicited wheal diameters of ≥ 3 mm to the saline/glycerol negative control (Supplementary Table 3). Therefore, each participant served as their own control to facilitate interpretation of SPT reactions to the three putative orphan allergens, two related proteins not included in allergen databases, histamine, and commercial extracts of four mold species. The SPT reactions to each of these materials were scored positive when their wheal diameter was ≥ 3 mm larger than the wheal diameter for the respective saline/glycerol negative control (Eigenmann and Sampson 1998). All SPT(+) reactions presented in Table 3 have been normalized relative to the variability in wheal reaction to the negative control to minimize reporting of false positive SPT reactions that might occur due to non-specific traumatic reactivity or dermographia (Hamilton and Adkinson 2003).

Only three of the 19 study participants that were SPT(+) to histamine elicited SPT(+) reactions to AaALDH or ChALDH, and none of these three showed SPT(+) reactions to their respective source organism, *A. alternata* or *C. herbarum*, raising a question about whether AaALDH and ChALDH are associated with allergic reactions to these mold organisms. Additionally, of three elicited SPT(+) reactions to AaALDH and ChALDH, two were elicited only by the low test dose, but not also by the higher test dose of the respective protein, inconsistent with these two proteins being allergenic. It is reasonable to conclude, therefore, that these few SPT(+) reactions to AaALDH and ChALDH, just like the single SPT(+) reaction to ZmALDH (a protein that is not listed in allergen databases), is a result of dermographia or non-specific local inflammatory reactions.

The published data suggesting that AaALDH and ChALDH are allergens is from a single study that used IgE sera from mold-allergic patients obtained from three local allergy clinics (Achatz et al. 1995). A total of 194 sera were tested, with 60% of the subjects having tested positive by radioallergosorbent (RAST) test to *A. alternata* and *C. herbarum*, the rest tested positive to a commercial mixed mold allergen RAST. In this Austrian study, out of 98 IgE serum samples that were reactive by immunoblot to *A. alternata* extracts, only two of these IgE sera were reactive to AaALDH (Alt a 10). This low level of IgE reactivity to AaALDH appears to be consistent with the present study’s SPT results in which reactivity to AaALDH was no different than reactivity to ZmALDH, a protein that is not listed in allergen databases. Likewise, in the present study reactivity of AaALDH was less than the SPT reactivity to *C. albicans*, a yeast that is not associated with elicitation of clinical allergies but is known to cause inflammatory sensitization consistent with SPT(+) reactions (Fukutomi and Taniguchi 2015).

In the same study (Achatz et al. 1995), IgE sera from 62 patients were reactive by immunoblot to *C. herbarum* extracts, with 22 (36%) of these IgE sera reactive to ChALDH (Cla h 3). However, the results with ChALDH in the present SPT studies do not support a conclusion that ChALDH is an allergen for the same reasons as mentioned above for AaALDH. The fact that the present SPT results were unable to confirm that ChALDH is an allergen, suggests the need for further study of whether or not ChALDH is allergenic.

Unlike the SPT results for the two ALDH proteins, 10 of the 19 validated SPT participants were SPT(+) to ChMDH, making this orphan allergen the most reactive material tested in this SPT study and producing results consistent with the report (Simon-Nobbe et al. 2006) that supported inclusion of ChMDH in allergen databases. Five of the six individuals that were SPT(+) to *C. herbarum* were SPT(+) to ChMDH, with four mold-allergic individuals reactive at both test doses of ChMDH. However, the other 50% of the individuals SPT(+) to ChMDH were not SPT(+) to the commercial extract of *C. herbarum*, a result most likely attributable to variability in SPT reactivity to commercial mold extracts (Simon-Nobbe et al. 2008). The study that first identified ChMDH as a putative allergen from *C. herbarum* found that 12 (57%) of 21 individuals allergic to *C. herbarum* had IgE sera reactive to ChMDH (Simon-Nobbe et al. 2006), leading the authors to conclude that ChMDH is the major allergenic protein for *C. herbarum*. This report also included an image of positive SPT reactivity to ChMDH for a single subject that was allergic to *C. herbarum*. However, although the present clinical results with ChMDH continue to support this protein being a major allergen for individuals allergic to *C. herbarum*, the structural studies were unable to identify the immunologically reactive epitope(s) of ChMDH that are distinct from other MDH proteins that are not identified as allergens, warranting future, more detailed, structural research.

### Considerations for interpreting alignments with orphan allergens

The data regarding orphan allergens presented to this point suggest a re-consideration of whether all protein allergens in allergen sequence databases should be considered equally when assessing the allergenic potential of proteins under review for introduction into the diet. Databases of allergen sequences represent a spectrum of protein families, in terms of allergenic propensity. At one end of the spectrum of protein families in allergen databases is the Bet v 1 family, in which Bet v 1 is a strong sensitizer, and many other members are elicitors that display a continuum of cross-reactivity (Roulias et al. 2014; Blankestijn et al. 2017; Biedermann et al. 2019). At the other end of the spectrum is the ALDH protein family with > 117,000 total members, and yet only five members are reported as allergens (Achatz et al. 1995; Nakazawa et al. 2007; Cui et al. 2016; Huerta-Ocampo et al. 2020), most associated with exposure to fungi, such as *Alternaria* and *Cladosporium*. Additionally, unlike the robust allergic reactivity to Bet v 1 and related allergens, the present SPT results, combined with previously reported IgE serum screening (Achatz et al. 1995), consistently show single digit percentages of reactivity with AaALDH, and marginally greater with ChALDH.

The MDH protein family is another example on the end of the spectrum shared with ALDH. Only three of the thousands of MDH protein family members are reported as allergens, with ChMDH showing strong allergenic potential in this study and previously (Simon-Nobbe et al. 2006). The other two MDH proteins reported as allergens are actually isoforms of the same protein from *A. alternata* (Schneider et al. 2006), since they differ by only a single amino acid. Noteworthy is that the rest of > 6000 members of the MDH protein family are not reported as allergens. Therefore, this highly diverse spectrum of the prevalence of allergens in protein families, from the highly allergenic Bet v 1 family to the sparsely allergenic MDH family, underscores the complexities in drawing conclusions from bioinformatic analysis of protein sequences that are being considered for introduction into the diet (NAS 2016; Ribeiro et al. 2018; FDA 2019; Abdelmoteleb et al. 2021; De Marchi et al. 2021; Montanari et al. 2021).

Currently, scientists and regulators evaluating proteins in foods derived from modern biotechnology follow guidance found in Codex (2009). This guidance states that any expressed protein is considered a potential allergen if it exceeds a threshold of greater than 35% sequence identity in a window of at least 80 amino acids for any sequence in an allergen database. This threshold is meaningful for novel sequences aligning with the Bet v 1 family of proteins, since this approach will identify even distant homologs (and there is a reasonable hypothesis that they might cross-react). However, the Codex threshold is far less informative when applied to alignments with orphan allergens, such as AaALDH, ChALDH and ChMDH. If an expressed protein meets the threshold of 35% identity in an 80 amino acid window with allergenic orphans, such as ALDH and MDH, it also meets the threshold with many thousands of family members that have never been identified as allergens. Many of the family members not identified as allergens have significant opportunity for human exposure, as discussed above for *Penicillium camemberti*, *Botrytis cinerea*, and *Baudoinia panamerican* (Simon-Nobbe et al. 2008; Twaroch et al. 2015; Williams et al. 2016). However, the vast majority of proteins in the ALDH and MDH families, while sharing much higher levels of identity with putative allergenic proteins, such as ChALDH, AaALDH and ChMDH, are present in species that have a long history of safe consumption as foodstuffs and/or from the environment. For this latter situation related to these putative orphan allergens, the end result of using a single bioinformatics threshold to identify potential allergens leads to a false positive conclusion that the expressed protein under review is likely to be an allergen. Negative results from additional testing, such as IgE-binding studies or SPT, are required before it’s possible to reverse the conclusion that “…IgE cross-reactivity between the newly expressed protein and a known allergen should be considered a possibility when there is more than 35 percent identity in a segment of 80 or more amino acids…” (Codex 2009). Therefore, while the inclusion criteria of sequences in databases of allergenic proteins should cast a broad net, the evidence supporting the conclusion that the database member is an allergen, and its relationship to other allergens and other non-allergenic family members, must be taken into consideration when interpreting alignment data.

In summary, two of the three orphan allergens in this study, AaALDH and ChALDH, did not elicit SPT(+) reactions consistent with inclusion in allergen databases, like COMPARE (2022). By comparison, ChMDH elicited SPT(+) reactions consistent with previously published results that identified it as an allergen. The present study, however, was unable to identify structural feature(s) of any of these putative orphan allergens suggestive that the feature(s) are the immunologically reactive epitope(s) that are distinct from other members of these two large protein families, ALDH and MDH, that are not identified as allergens. With the ubiquity of large protein families, such as ALDH and MDH, in which most protein members are not included in allergen databases, bioinformatic methods designed to assess protein allergenicity need to advance beyond the current “one size fits all” approach. Updates to bioinformatic methods that bring to bear full knowledge related to the complete range of allergens, from pan allergens to orphan allergens, along with their non-allergenic family members, would facilitate more effective selection of safe newly expressed food proteins.

## Supplementary Information

Below is the link to the electronic supplementary material.

Supplementary file1 (DOCX 4705 kb)

## Abbreviations

ALDH: Aldehyde dehydrogenase
IgE: Immunoglobulin E
MCL: Markov Cluster algorithm
MDH: Mannitol dehydrogenase
PDB: Protein Data Bank
SDR: Short-chain dehydrogenase/reductase
SPT: Skin prick testing

## References

[CR1] Abdelmoteleb M, Zhang C, Furey B, Kozubal M, Griffiths H, Champeaud M, Goodman RE (2021) Evaluating potential risks of food allergy of novel food sources based on comparison of proteins predicted from genomes and compared to www.AllergenOnline.org. Food Chem Toxicol 147:111888. 10.1016/j.fct.2020.111888 10.1016/j.fct.2020.111888 33276067

[CR2] ACAAI (2021) Allergy facts. The American College of Allergy, Asthma & Immunology. https://acaai.org/allergies/allergies-101/facts-stats/. Accessed 02 Oct 2021

[CR3] Achatz G, Oberkofler H, Lechenauer E, Simon B, Unger A, Kandler D, Ebner C, Prillinger H, Kraft D, Breitenbach M (1995). Molecular cloning of major and minor allergens of *Alternaria alternata* and *Cladosporium herbarum*. Mol Immunol.

[CR4] Adams PD, Afonine PV, Bunkóczi G, Chen VB, Davis IW, Echols N, Headd JJ, Hung LW, Kapral GJ, Grosse-Kunstleve RW, McCoy AJ, Moriarty NW, Oeffner R, Read RJ, Richardson DC, Richardson JS, Terwilliger TC, Zwart PH (2010). PHENIX: a comprehensive Python-based system for macromolecular structure solution. Acta Crystallogr D Biol Crystallogr.

[CR5] AllergenOnline (2021) AllergenOnline. Home of the FARRP allergen protein database. http://allergenonline.com/. Accessed 02 Oct 2021

[CR6] Altschul SF, Gish W, Miller W, Myers EW, Lipman DJ (1990). Basic local alignment search tool. J Mol Biol.

[CR7] Ansotegui IJ, Melioli G, Canonica GW, Caraballo L, Villa E, Ebisawa M, Passalacqua G, Savi E, Ebo D, Gómez RM, Luengo Sánchez O, Oppenheimer JJ, Jensen-Jarolim E, Fischer DA, Haahtela T, Antila M, Bousquet JJ, Cardona V, Chiang WC, Demoly PM, DuBuske LM, Ferrer Puga M, Gerth van Wijk R, González Díaz SN, Gonzalez-Estrada A, Jares E, Kalpaklioğlu AF, Kase Tanno L, Kowalski ML, Ledford DK, Monge Ortega OP, Morais Almeida M, Pfaar O, Poulsen LK, Pawankar R, Renz HE, Romano AG, Rosário Filho NA, Rosenwasser L, Sánchez Borges MA, Scala E, Senna GE, Sisul JC, Tang MLK, Thong BY, Valenta R, Wood RA, Zuberbier T (2020). IgE allergy diagnostics and other relevant tests in allergy, a World Allergy Organization position paper. World Allergy Organ J.

[CR8] Biedermann T, Winther L, Till SJ, Panzner P, Knulst A, Valovirta E (2019) Birch pollen allergy in Europe. Allergy 74:1237–1248. 10.1111/all.13758 10.1111/all.13758 30829410

[CR9] Blankestijn MA, Knulst AC, Knol EF, Le TM, Rockmann H, Otten HG, Klemans RJB (2017). Sensitization to PR-10 proteins is indicative of distinctive sensitization patterns in adults with a suspected food allergy. Clin Transl Allergy.

[CR10] Carr WW, Martin B, Howard RS, Cox L, Borish L (2005). Comparison of test devices for skin prick testing. J Allergy Clin Immunol.

[CR11] Codex (2009) Foods derived from modern biotechnology. FAO and WHO. http://www.fao.org/3/a-a1554e.pdf. Accessed 8 Jan 2017

[CR12] COMPARE (2022) COMprehensive Protein Allergen REsource. https://comparedatabase.org/. Accessed 01 Jun 2022

[CR13] Cui Y, Yu L, Teng F, Zhang C, Wang N, Yang L, Zhou Y (2016). Transcriptomic/proteomic identification of allergens in the mite *Tyrophagus putrescentiae*. Allergy.

[CR14] De Marchi L, Wangorsch A, Zoccatelli G (2021). Allergens from edible insects: cross-reactivity and effects of processing. Curr Allergy Asthma Rep.

[CR15] De Vouge MW, Thaker AJ, Zhang L, Muradia G, Rode H, Vijay HM (1998). Molecular cloning of IgE-binding fragments of *Alternaria alternata* allergens. Int Arch Allergy Immunol.

[CR16] Díaz-Sánchez Á, González-Segura L, Mújica-Jiménez C, Rudiño-Piñera E, Montiel C, Martínez-Castilla LP, Muñoz-Clares RA (2012). Amino acid residues critical for the specificity for betaine aldehyde of the plant ALDH10 isoenzyme involved in the synthesis of glycine betaine. Plant Physiol.

[CR17] EFSA (2006) Guidance document for the risk assessment of genetically modified plants and derived food and feed by the Scientific Panel on Genetically Modified Organisms (GMO)—including draft document updated in 2008. EFSA Journal 4:99. 10.2903/j.efsa.2006.99

[CR18] EFSA (2011) Guidance for risk assessment of food and feed from genetically modified plants. EFSA J 9:2150. 10.2903/j.efsa.2011.2150 10.2903/j.efsa.2011.2150 PMC13177335 42148424

[CR19] Eigenmann PA, Sampson HA (1998). Interpreting skin prick tests in the evaluation of food allergy in children. Pediatr Allergy Immunol.

[CR20] El-Gebali S, Mistry J, Bateman A, Eddy SR, Luciani A, Potter SC, Qureshi M, Richardson LJ, Salazar GA, Smart A, Sonnhammer ELL, Hirsh L, Paladin L, Piovesan D, Tosatto SCE, Finn RD (2019). The Pfam protein families database in 2019. Nucleic Acids Res.

[CR21] Emsley P, Lohkamp B, Scott WG, Cowtan K (2010). Features and development of *Coot*. Acta Crystallogr D Biol Crystallogr.

[CR22] Enright AJ, Van Dongen S, Ouzounis CA (2002). An efficient algorithm for large-scale detection of protein families. Nucleic Acids Res.

[CR23] FDA (2019) FDA announces effective date for final rule adding soy leghemoglobin to list of color additives exempt from certification. US Food and Drug Administration. https://www.fda.gov/food/cfsan-constituent-updates/fda-announces-effective-date-final-rule-adding-soy-leghemoglobin-list-color-additives-exempt. Accessed 02 Oct 2021

[CR24] Ferreira F, Hawranek T, Gruber P, Wopfner N, Mari A (2004). Allergic cross-reactivity: from gene to the clinic. Allergy.

[CR25] Fisher M, Kroon JT, Martindale W, Stuitje AR, Slabas AR, Rafferty JB (2000). The X-ray structure of Brassica napus beta-keto acyl carrier protein reductase and its implications for substrate binding and catalysis. Structure.

[CR26] Fruchterman TMJ, Reingold EM (1991) Graph drawing by force-directed placement. Softw Pract Exp 21:1129–1164. 10.1002/spe.4380211102

[CR27] Fukutomi Y, Taniguchi M (2015). Sensitization to fungal allergens: resolved and unresolved issues. Allergol Int.

[CR28] González-Segura L, Riveros-Rosas H, Julián-Sánchez A, Muñoz-Clares RA (2015). Residues that influence coenzyme preference in the aldehyde dehydrogenases. Chem Biol Interact.

[CR29] Hamilton RG, Adkinson NF (2003). 23. Clinical laboratory assessment of IgE-dependent hypersensitivity. J Allergy Clin Immunol.

[CR30] Hauser M, Roulias A, Ferreira F, Egger M (2010). Panallergens and their impact on the allergic patient. Allergy Asthma Clin Immunol.

[CR31] Heinzerling L, Mari A, Bergmann KC, Bresciani M, Burbach G, Darsow U, Durham S, Fokkens W, Gjomarkaj M, Haahtela T, Bom AT, Wöhrl S, Maibach H, Lockey R (2013). The skin prick test - European standards. Clin Transl Allergy.

[CR32] Hörer S, Stoop J, Mooibroek H, Baumann U, Sassoon J (2001). The crystallographic structure of the mannitol 2-dehydrogenase NADP+ binary complex from *Agaricus bisporus*. J Biol Chem.

[CR33] Huerta-Ocampo J, Valenzuela-Corral A, Robles-Burgueño MDR, Guzmán-Partida AM, Hernández-Oñate M, Vázquez-Moreno L, Pavón-Romero GF, Terán LM (2020). Proteomic identification of allergenic proteins in red oak (*Quercus rubra*) pollen. World Allergy Organ J.

[CR34] Jenkins JA, Griffiths-Jones S, Shewry PR, Breiteneder H, Mills EN (2005). Structural relatedness of plant food allergens with specific reference to cross-reactive allergens: an in silico analysis. J Allergy Clin Immunol.

[CR35] Kavanagh KL, Jörnvall H, Persson B, Oppermann U (2008). Medium- and short-chain dehydrogenase/reductase gene and protein families: the SDR superfamily: functional and structural diversity within a family of metabolic and regulatory enzymes. Cell Mol Life Sci.

[CR36] Kessenich C, Silvanovich A (2021) Challenges of automation and scale: bioinformatics and the evaluation of proteins to support genetically modified product safety assessments. J Invertebr Pathol:107587. 10.1016/j.jip.2021.107587 33838205

[CR37] Kopečny D, Končitíková R, Tylichová M, Vigouroux A, Moskalíková H, Soural M, Šebela M, Moréra S (2013). Plant ALDH10 family: identifying critical residues for substrate specificity and trapping a thiohemiacetal intermediate. J Biol Chem.

[CR38] Korasick DA, Končitíková R, Kopečná M, Hájková E, Vigouroux A, Moréra S, Becker DF, Šebela M, Tanner JJ, Kopečný D (2019). Structural and biochemical characterization of aldehyde dehydrogenase 12, the last enzyme of proline catabolism in plants. J Mol Biol.

[CR39] Lang BS, Gorren AC, Oberdorfer G, Wenzl MV, Furdui CM, Poole LB, Mayer B, Gruber K (2012). Vascular bioactivation of nitroglycerin by aldehyde dehydrogenase-2: reaction intermediates revealed by crystallography and mass spectrometry. J Biol Chem.

[CR40] Matsui EC, Abramson SL, Sandel MT (2016). Indoor environmental control practices and asthma management. Pediatrics.

[CR41] McClain S (2017). Bioinformatic screening and detection of allergen cross-reactive IgE-binding epitopes. Mol Nutr Food Res.

[CR42] McClerklin SA, Lee SG, Harper CP, Nwumeh R, Jez JM, Kunkel BN (2018). Indole-3-acetaldehyde dehydrogenase-dependent auxin synthesis contributes to virulence of *Pseudomonas syringae* strain DC3000. PLoS Pathog.

[CR43] McCoy AJ, Grosse-Kunstleve RW, Adams PD, Winn MD, Storoni LC, Read RJ (2007). Phaser crystallographic software. J Appl Crystallogr.

[CR44] Minor W, Cymborowski M, Otwinowski Z, Chruszcz M (2006). HKL-3000: the integration of data reduction and structure solution–from diffraction images to an initial model in minutes. Acta Crystallogr D Biol Crystallogr.

[CR45] Montanari F, de Moura AP, Cunha LM (2021). Production and commercialization of insects as food and feed identification of the main constraints in the European Union.

[CR46] Moretti A, Li J, Donini S, Sobol RW, Rizzi M, Garavaglia S (2016). Crystal structure of human aldehyde dehydrogenase 1A3 complexed with NAD(+) and retinoic acid. Sci Rep.

[CR47] Muraro A, Werfel T, Hoffmann-Sommergruber K, Roberts G, Beyer K, Bindslev-Jensen C, Cardona V, Dubois A, duToit G, Eigenmann P, Fernandez Rivas M, Halken S, Hickstein L, Høst A, Knol E, Lack G, Marchisotto MJ, Niggemann B, Nwaru BI, Papadopoulos NG, Poulsen LK, Santos AF, Skypala I, Schoepfer A, Van Ree R, Venter C, Worm M, Vlieg-Boerstra B, Panesar S, de Silva D, Soares-Weiser K, Sheikh A, Ballmer-Weber BK, Nilsson C, de Jong NW, Akdis CA (2014). EAACI food allergy and anaphylaxis guidelines: diagnosis and management of food allergy. Allergy.

[CR48] Nakazawa T, Satinover SM, Naccara L, Goddard L, Dragulev BP, Peters E, Platts-Mills TA (2007). Asian ladybugs (Harmonia axyridis): a new seasonal indoor allergen. J Allergy Clin Immunol.

[CR49] NAS (2016) Genetically engineered crops: experiences and prospects, National Academies Press 28230933

[CR50] Nüss D, Goettig P, Magler I, Denk U, Breitenbach M, Schneider PB, Brandstetter H, Simon-Nobbe B (2010). Crystal structure of the NADP-dependent mannitol dehydrogenase from Cladosporium herbarum: Implications for oligomerisation and catalysis. Biochimie.

[CR51] Pfam (2021a) Family: Aldedh (PF00171). http://pfam.xfam.org/family/PF00171 Accessed 02 Oct 2021a

[CR52] Pfam (2021b) Pfam 34.0. http://pfam.xfam.org/ Accessed 17 May 2011

[CR53] Ribeiro JC, Cunha LM, Sousa-Pinto B, Fonseca J (2018). Allergic risks of consuming edible insects: a systematic review. Mol Nutr Food Res.

[CR54] Roulias A, Pichler U, Hauser M, Himly M, Hofer H, Lackner P, Ebner C, Briza P, Bohle B, Egger M, Wallner M, Ferreira F (2014). Differences in the intrinsic immunogenicity and allergenicity of Bet v 1 and related food allergens revealed by site-directed mutagenesis. Allergy.

[CR55] Schneider PB, Denk U, Breitenbach M, Richter K, Schmid-Grendelmeier P, Nobbe S, Himly M, Mari A, Ebner C, Simon-Nobbe B (2006). Alternaria alternata NADP-dependent mannitol dehydrogenase is an important fungal allergen. Clin Exp Allergy.

[CR56] Shortall K, Djeghader A, Magner E, Soulimane T (2021). Insights into aldehyde dehydrogenase enzymes: a structural perspective. Front Mol Biosci.

[CR57] Sicherer SH, Sampson HA (2018). Food allergy: A review and update on epidemiology, pathogenesis, diagnosis, prevention, and management. J Allergy Clin Immunol.

[CR58] Simon-Nobbe B, Denk U, Pöll V, Rid R, Breitenbach M (2008). The spectrum of fungal allergy. Int Arch Allergy Immunol.

[CR59] Simon-Nobbe B, Denk U, Schneider PB, Radauer C, Teige M, Crameri R, Hawranek T, Lang R, Richter K, Schmid-Grendelmeier P, Nobbe S, Hartl A, Breitenbach M (2006). NADP-dependent mannitol dehydrogenase, a major allergen of Cladosporium herbarum. J Biol Chem.

[CR60] Theocharidis A, van Dongen S, Enright AJ, Freeman TC (2009). Network visualization and analysis of gene expression data using BioLayout Express(3D). Nat Protoc.

[CR61] Twaroch TE, Curin M, Valenta R, Swoboda I (2015). Mold allergens in respiratory allergy: from structure to therapy. Allergy Asthma Immunol Res.

[CR62] van Dongen S, Abreu-Goodger C (2012) Using MCL to extract clusters from networks. In: van Helden J, Toussaint A, Thieffry D (ed) Bacterial molecular networks: methods and protocols, New York, NY, Springer New York**,** 281–295 10.1007/978-1-61779-361-5_15 22144159

[CR63] Van Dongen SM (2000) Graph clustering by flow simulation. https://dspace.library.uu.nl/bitstream/handle/1874/848/full.pdf?sequence=1. Accessed

[CR64] Venkatesan R, Sah-Teli SK, Awoniyi LO, Jiang G, Prus P, Kastaniotis AJ, Hiltunen JK, Wierenga RK, Chen Z (2014). Insights into mitochondrial fatty acid synthesis from the structure of heterotetrameric 3-ketoacyl-ACP reductase/3R-hydroxyacyl-CoA dehydrogenase. Nat Commun.

[CR65] Williams PB, Barnes CS, Portnoy JM (2016). Innate and adaptive immune response to fungal products and allergens. J Allergy Clin Immunol Pract.

