# Supplementary material for: Biochemical and clinical studies of putative allergens to assess what distinguishes them from other non-allergenic proteins in the same family
Source: Transgenic Res. 2022 Aug 8;31(4-5):507–24. doi: 10.1007/s11248-022-00316-8 (PMC9489553; doi:10.1007/s11248-022-00316-8)

**Biochemical and clinical studies of putative allergens to assess what distinguishes them from other non-allergenic proteins in the same family**

**Transgenic Research**

Kevin C. Glenn^1*^, Andre Silvanovich^1^, Soon Goo Lee^2,4^, Aron Allen^2^, Stephanie Park^3^, S. Eliza Dunn^1^, Colton Kessenich^1^, Chen Meng^1^, John L. Vicini^1^, Joseph M. Jez^2^

^1^Bayer Crop Science, 700 Chesterfield Pkwy W, Chesterfield, MO 63017 USA; ^2^Department of Biology, Washington University, CB 1137, One Brookings Dr., St. Louis, MO 63130 USA; ^3^Allergy and Asthma Care of St. Louis, 8888 Ladue Road, Suite 105, St. Louis, MO 63124 USA; ^4^Department of Chemistry and Biochemistry, University of North Carolina Wilmington, Wilmington, NC 28403 USA.

**Corresponding Author**: John L. Vicini (john.vicini@bayer.com)

**Supplementary Information**

**Supplementary Table 1. Protein structure and sequence comparison.** (A) Identification of ChALDH structurally related proteins and pairwise structural comparisons were performed using the DALI server (<http://ekhidna.biocenter.helsinki.fi/dali_server/>) and the NCBI standard protein BLAST against Protein Data Bank (PDB) proteins as described in **Fig. 2**. (B) Identification of the ChMDH structure-related proteins and pairwise structural comparisons were performed as described above and corresponds with **Fig. 3.** Each table notes the PDB code, Z-score, and r.m.s.d for the pair-wise alignment over the number of residues aligned (lali) and total residues (nres), as well as the percent amino acid sequence identity (%id) between the template and target structures.

| **Supplemental Table 2. Criteria for enrollment of clinical SPT study participants.** | | |
| --- | --- | --- |
| **Inclusion Criteria**  *(all must be met)* | ≥ 18 years of either sex and any race | |
|  | Physician diagnosed allergy to mold that includes a current or previously shown positive skin prick test (SPT) response to mold (diameter of wheal 3 mm or greater than a negative control) AND physician established convincing clinical history of allergy-related symptoms to mold. | |
|  | Signed informed consent | |
|  | Adherence to the standard office procedure for preparation for SPT testing (e.g., discontinued usage of allergy and asthma-related medications for ≥ 5 days: antihistamines, oral and/or inhaled steroid medications (e.g., fluticasone). | |
|  | | |
| **Exclusion Criteria**  *(none can be met)* | Inability to discontinue allergy and/or asthma-related medications for ≥ 5 days prior to SPT testing (e.g., antihistamines oral and/or inhaled steroids such as fluticasone). | |
|  | Diagnosis of any of the following: | - uncontrolled hypertension |
|  |  | - active eosinophilic gastritis |
|  |  | - severe persistent and/or refractory asthma |
|  |  | - history of intubation for asthma |
|  |  | - severe atopic dermatitis (as assessed by a Three Item Severity score of ≥ 6) |
|  | Any use of beta-blockers, angiotensin converting enzyme (ACE) inhibitors, angiotensin-receptor blockers (ARB) | |
|  | Other significant medical conditions which, in the opinion of the clinical principal investigator, makes the patient unsuitable for inclusion | |
|  | Pregnancy | |

**Supplementary Table 3. Summary of clinical skin prick testing (SPT).**  SPT results are shown for all 27 study participants. The (*) indicates dermographia. The diameter (mm) of each wheal/flare reaction is shown. For each subject, SPT reactions were scored as positive when the wheal diameter was ≥ 3mm larger than the reaction to the negative control (highlighted in yellow). SPT wheal diameter reactions ≥ 3mm larger than the reaction to the negative control for participants that were SPT(-) to histamine are grayed-out, since they were not included in Table 3.

**
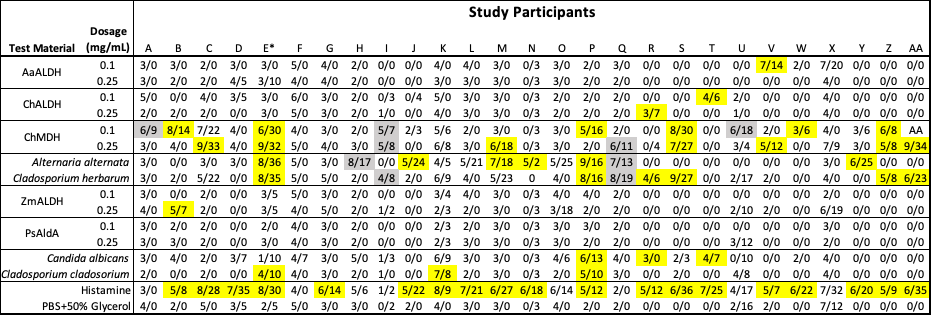
**

**Supplementary Figure 1. Expression and purification of ChALDH, AaALDH, and ChMDH.** Size-exclusion chromatograms (A_280nm_) are shown for (A) ChALDH, (B) AaALDH, and (C) ChMDH. The inset in each panel shows SDS-PAGE analysis of ChALDH, AaALDH, and ChMDH, respectively. Samples were stained for total protein using Coomassie Blue. Numbers correspond to the indicated molecular mass markers (kDa). Lanes are as follows: molecular mass markers and size-exclusion purified protein used for protein crystallization and/or SPT.


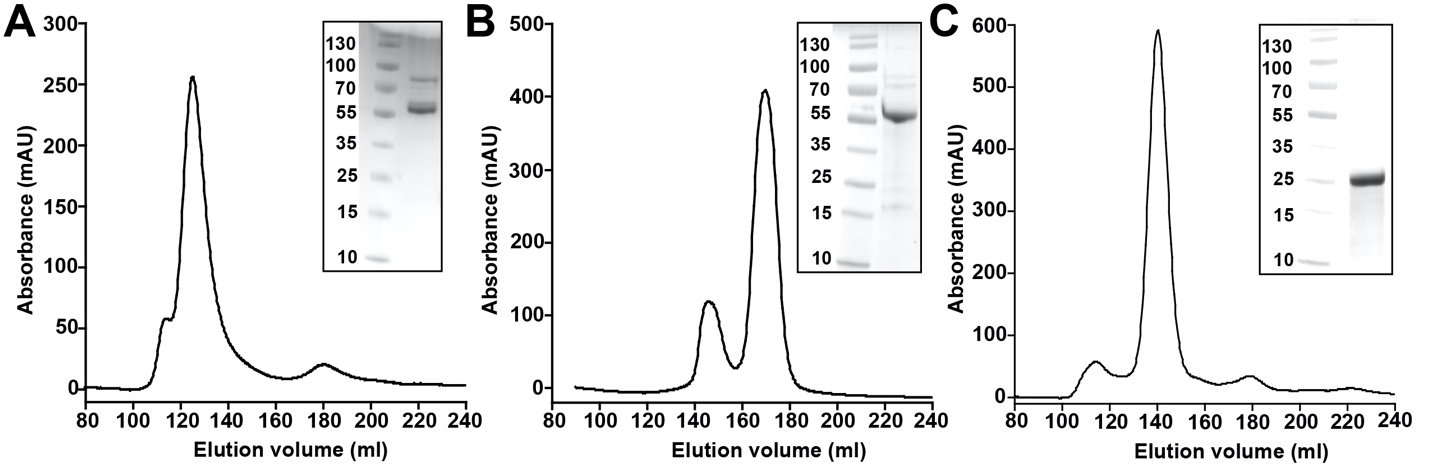


**Supplementary Figure 2. Domain architecture and active site of ChALDH.**  (A) The UniProt aldehyde dehydrogenase (ALDH) domains (colored salmon in ChALDH and purple in AaALDH, respectively), NAD(P) binding site (blue triangles), and catalytic residues (red triangles) of *C. herbarum* (CAA55072.2) and *A. alternata* (CAA55071.2) were predicted using InterPro79.0. (B) The secondary structure of ChALDH is shown above the amino acid sequences with regions corresponding to α-helices and β-strands shown as gold tubes and blue arrows, respectively. (C) Topology of ChALDH showing the domains described in **Fig. 2** with the same coloring as above and secondary structure features numbered. (D) Secondary structure features of the ChALDH monomer. The α‑helices and β-strands of ChALDH are shown as gold cylinders and blue arrows, respectively. The domain organization of the monomer is highlighted as follows: catalytic domain (purple), NAD(P)(H) binding domain (salmon), and the oligomerization domain (green). (E) Electron density for a representative α-helix (amino acid residues from 221 to 230 in monomer A) in the ChALDH structure is shown as a 2F_o_-F_c_ omit map (1.6 σ). (F) Amino acid residues forming interactions were predicted with the computationally docked NADP^+^ in the ChALDH active site using Autodock Vina. Residue side-chains interacting with NADP^+^ (salmon) are shown as stick-renderings. Hydrogen bond interactions between the amino acid residues and ligands are shown as yellow dotted lines. The catalytic cysteine is labeled.


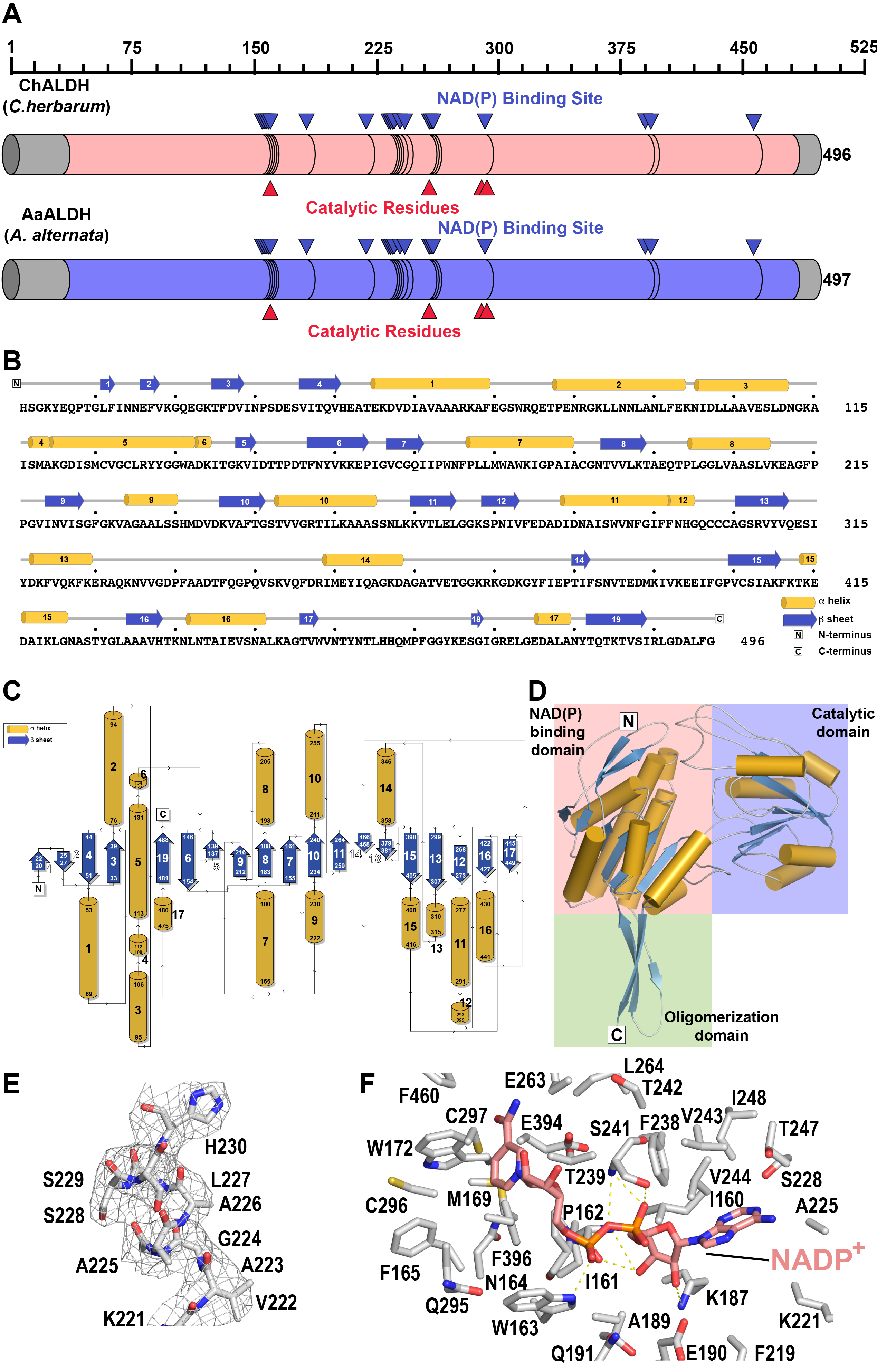


**Supplementary Figure 3. Domain architecture and active site of ChMDH.** (A) The UniProt short-chain dehydrogenase/reductase (SDR) domain (colored green in ChMDH), NAD(P)(H) binding site (blue triangles), and catalytic residues (red triangles) of *C. herbarum* (P0C0Y5) were predicted using InterPro79.0. (B) Secondary structure features of a ChMDH monomer are colored, as follows: α-helices and β-strands are shown as gold cylinders and blue arrows, respectively. NADP^+^ is shown as a space-filling model. (C) Electron density for NADP^+^ in the ChMDH•NADP^+^ structure is shown as a 2F_o_-F_c_ omit map (1.6 σ). (D) The secondary structure of ChMDH is shown above the amino acid sequences with regions corresponding to α-helices and β-strands shown as gold tubes and blue arrows, respectively. N- and C- termini are labeled. (E) Topology of ChMDH showing the domains described in Fig. 3 with the same coloring as above and secondary structure features numbered. (F) Amino acid side-chains of residues interacting with NADP^+^ (orange) are shown as stick-renderings. Hydrogen bond interactions between the amino acid residues and ligands are shown as yellow dotted lines. The catalytic cysteine is labeled. Side-chains of residues interacting with NADP^+^ (orange) are shown as stick-renderings. Waters interacting with the cofactor are shown as red spheres. Hydrogen bonds are indicated by yellow dotted lines.


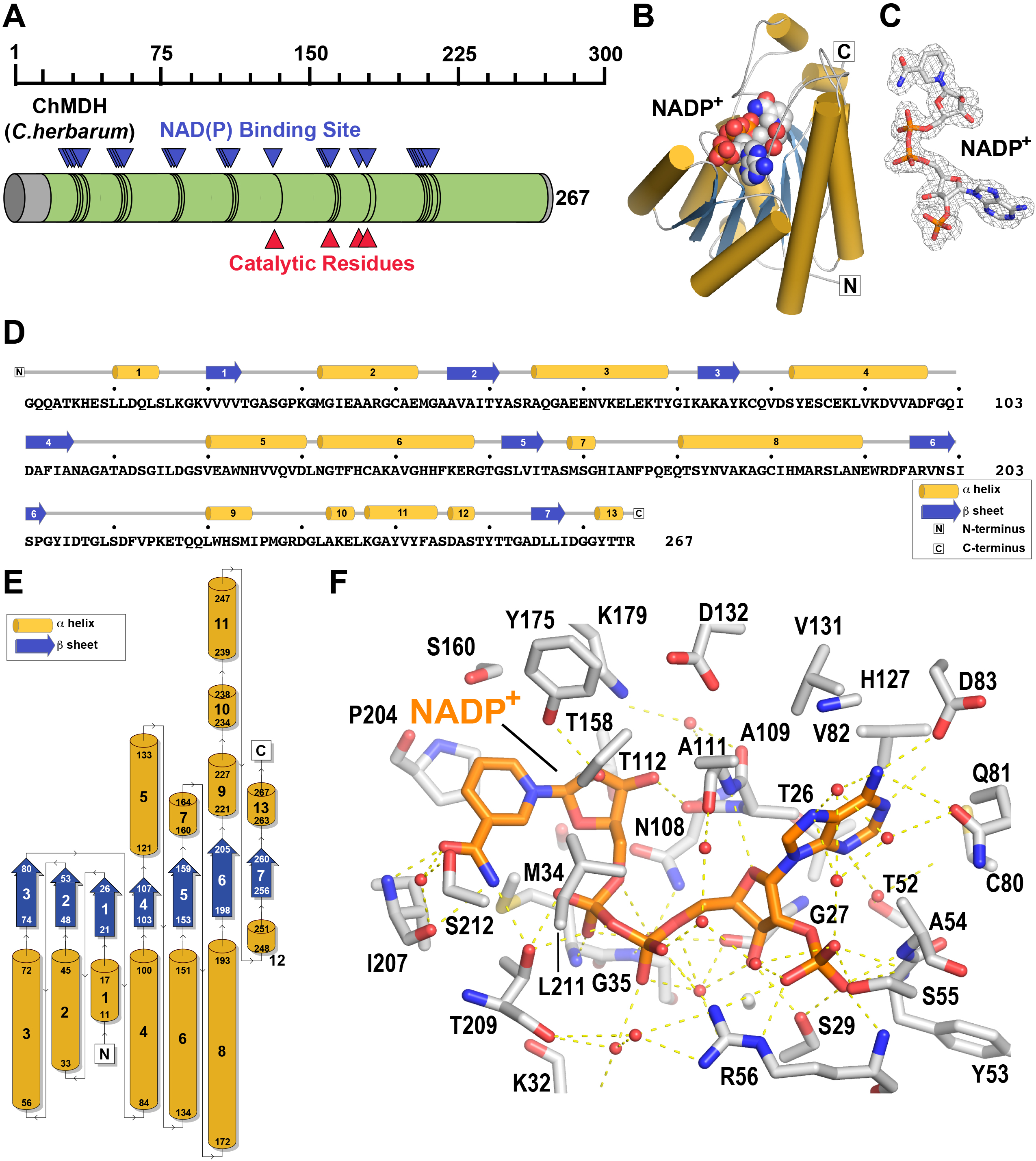

Supplement: Supplementary file 1 — Supplementary file1 (DOCX 4705 kb) [file 11248_2022_316_MOESM1_ESM.docx]
